# Optical Third Harmonic Generation Using Nickel Nanostructure-Covered Microcube Structures

**DOI:** 10.3390/ma11040501

**Published:** 2018-03-27

**Authors:** Yoichi Ogata, Anatoliy Vorobyev, Chunlei Guo

**Affiliations:** The Institute of Optics, University of Rochester, Hutchison Road 275, Rochester, NY 14627, USA; avorobye@ur.rochester.edu

**Keywords:** Ni, THG, nonlinearity, plasmon, lightning rod

## Abstract

We investigated the optical third harmonic generation (THG) signal from nanostructure-covered microcubes on Ni. We found that the hierarchical structures greatly change the third-order optical nonlinearity of the metallic surface. While the symmetry and lightning rod (LR) effects on microstructures did not significantly influence the THG, the localized surface plasmon (LSP) effect on the nanostructures enhanced it. By removing the nanostructures on the hierarchical structures, THG intensity could be strongly suppressed. In the present paper, we also discuss the mechanism that enhances THG in nano/micro structures.

## 1. Introduction

Optical third harmonic generation (THG) is a complicated coherent nonlinear optical process [1]. In general, nonlinear waves are forbidden for centrosymmetric systems [1,2,3,4]. However, it is clear that the magnitude of the THG signal is dependent on the specific structure of the sample. THG processes are useful for various applications, such as autocorrelators [5] and nonlinear imaging [6]. Since nonlinear processes through photon–photon interactions are intrinsically weak, studies on the possibility of enhancing the nonlinear efficiency are essential [1,3,4].

The enhancement of THG responses by surface plasmons (SPs) has been reported among many researchers [7,8,9]. SPs are coherent electron oscillations that exist at the metal surface [10]. Since THG intensity is proportional to the third power of the incident light intensity [1], the SP-assisted electric field enhancement at the surface areas greatly enhances THG light strength. The magnitude of this enhancement is different from that of optical second harmonic generation (SHG).

Recently, we produced nanostructure-covered laser-induced periodic surface structures (NC-LIPSSs) [11,12] and investigated their SHG [13,14,15]. We found that NC-LIPSSs could significantly modify the second-order nonlinearity of the metallic surfaces. The micro-scale grooves and the nanostructures on the NC-LIPSSs could influence not only symmetry but also SPs [13,14,15].

In spite of the systematic study on the SP-assisted SHG processes in nano/micro hierarchical structures [13,14,15], a systematic study on the SP-assisted THG processes of nano/micro hierarchical structures is currently lacking. Nano- and micro structures both play important roles for generating SPs. Therefore, a sufficient investigation of the dual enhancement effect of nano/micro hierarchical structures is needed to fully understand the potential applications these structures might have in nonlinear plasmon devices. One interesting component of the recently obtained structure, the nanostructure-covered microcube (NC-MC) [14], is their dominant feature. The different sizes of the surface structure excite different SPs. The micro-cubes (MCs) on the NC-MC surface lead to lightning rod (LR) effects. Meanwhile, the nanostructures developed on surface layer of the hierarchical structure can also excite localized SPs (LSPs). Here, LR effects induced at the corners of cubes lead to large local field enhancements, evidenced by much electromagnetic (EM) wave simulation [16,17,18]. Strong local fields at the tips (corners) enhance the SHG by over two orders of magnitude compared with a nonsharp reference [19]. By sufficiently studying the SPs excited by structures of different sizes and the SPs combined with hierarchical structures, we can control the THG efficiency in nano/micro structures. In this study, we observe THG signals from Ni NC-MCs created via the laser ablation method. We will discuss how their THG properties relate to their nano/micro structure.

## 2. Materials and Methods

**Figure 1:**
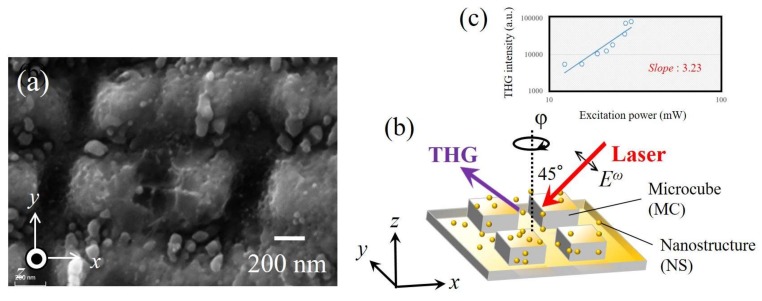
(**a**) A scanning electron microscope (SEM) image of the nanostructure-covered microcube (NC-MC) structures on Ni from a planar view normal to the surface; (**b**) Schematic detailing of measurement conditions. The rotation angle *φ* is defined as the angle between the incident plane and the travel direction of beam. When *φ* = 0°, the incident light propagates along the *x*-axis direction; (**c**) Excitation power dependence of the third harmonic generation (THG) intensity from the NC-MC sample.

THG measurements on the NC-MCs on Ni, as seen in Figure 1a, were performed using an amplified femtosecond (fs) laser. Using an amplified fs laser system, two laser beams with different polarization directions (i.e., *x* and *y* directions) were focused on the Ni surface. Then, the two beams were controlled by a mechanical chopper and the number of pulses per pulse burst was adjusted to be 1. Namely, it can be said that the two beams alternately passed through the chopper pulse by pulse. Here, the laser fluence and incidence angle were set at 0.137 J/cm^2^ and 1–1.5° (≒normal to the substrate), respectively. By doing so, we succeeded in producing an NC-MCs sample on Ni. After fabrication, the surface morphologies of NC-MCs were monitored by a scanning electron microscope (SEM). The depth of the microstructures was measured by a ultra-violet (UV) laser microscope that has a lateral resolution of 200 nm and transverse resolution as high as 1 nm. Figure 1a shows the SEM image of the Ni NC-MCs. According to Figure 1a, the average periodicity Λ of the micro-cubes on Ni was 600 nm, and their depth *d* was ~100 nm. Moreover, the micro-cubes were covered with nanostructures. The fabrication method and other details of the sample have been reported elsewhere [14,15]. Figure 1b shows the arrangement of the THG intensity measurements performed on the NC-MC sample. An amplified fs laser (60 fs, 1.2 W, 1 kHz repetition rate) emitting at ~800 nm with the *s*-polarization was focused onto the NC-MCs sample with a 45° incidence. The *s*-polarization is parallel to the *y*-direction. We observed the dependence of the THG intensity on laser polarization. THG with 266 nm was directed through a color filter and a band-pass filter (FGUV5) to cut the surplus fundamental light. An output light was set to the *s*-polarization. By acknowledging the influence of hyper-Rayleigh scattering (HRS) [20], the generated THG photons were collected through a large N.A. lens. Then, the THG signal was detected using a photomultiplier tube (PMT). 

## 3. Results

### 3.1. Definition of THG Intensity for Ni NC-MCs

The *s*-polarized THG intensity |*E_s_* (3*ω*)|^2^ {= *I_s_*(3*ω*)} with the $\chi_{YYYY}^{\left(3\right)}$ element is given as:

(1) $$|E_{s}^{R}\left(3\omega \right){|}^{2}\propto |F_{Y}\left(3\omega \right)\chi_{YYYY}^{\left(3\right)}F_{Y}{\left(\omega \right)}^{3}E_{Y}{\left(\omega \right)}^{3}{|}^{2}$$

where third-order nonlinear susceptibility *χ*^(3)^*_ijkl_* is a fourth rank tensor. The indices *ijkl* are summed over the three directions of laser polarization *x*, *y*, and *z*. The coordinate system is oriented so that the *x* and *y* coordinates are in the plane and the *z* coordinate is in the direction normal to the substrate surface [14]. *F_Y_*(*ω*) is the Fresnel factor at the fundamental frequency for the *y* direction, and *F_Y_*(3*ω*) is the Fresnel factor at the THG frequency along the *y* direction. The relation as *E_Y,loc_* = *F_Y_*(*ω*)*E_Y_*(*ω*) holds for the local electric field *E_loc_*. In the case of nanostructures, *F_Y_*(*ω*) can depend on both LSP and LR effects [21]. On the other hand, *F_Y_*(3*ω*) is expected to originate only from the LR effect because of no resonance. Either way, Equation (1) expresses that the *s*-polarized THG intensity |*E_s_*(3*ω*)|^2^ depends on the nonlinear susceptibility *χ* and the *y*-directed local field *E_Y,loc_* of the electric field component. 

### 3.2. THG Signal Intensity for Ni NC-MCs

We first investigated the output THG power dependence on fundamental frequency in the NC-MC sample. The power dependence of the THG intensity fits a slope of 3.23 when plotted on a log-log scale as shown in Figure 1c, which copes well with the third-order nature of the light emission.

**Figure 2:**
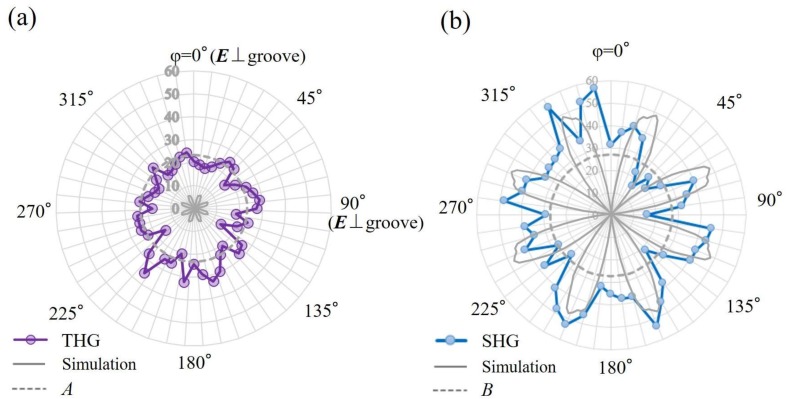
Angular (**a**) THG and (**b**) second harmonic generation (SHG) signal intensity. The angular nonlinear intensity of the NC-MCs as a function of the *φ*. The solid curve results from a simulation curve implanting a calculation by symmetrical parameters and local electric fields.

In order to investigate the origin of the THG, the *χ*^(3)^ of the microstructure surface was analyzed. The *χ*^(3)^ is sensitive to the symmetry of the structure. Since the structure of MCs in a tetragonal system is characterized by *C_4v_* symmetry with four mirror planes, 21 or 11 nonlinear susceptibility elements are permitted [1]. Under *s*-polarization configuration, the *χ*^(3)^*_YYYY_* should be accepted. If we assume that the *χ*^(3)^*_YYYY_* is dominated to the *s*-in/*s*-out polarization combination, the THG should be forbidden for *φ* = 0°, 45°, and 90° when *φ* ranges from 0° to 90°. In conclusion, we found that the *χ*^(3)^*_YYYY_* dependence on a sin(4*φ*) function is the main factor that modulates the THG emission from the MCs. The THG related to the symmetry for the MCs was then fitted by a sin^2^(4*φ*) function, and this trend is the same as the one found for the SHG case [14,15]. On the other hand, the nanostructures contain nanoparticles with strong quadrupolar resonances, and they should provide the fixed nonlinear intensities independent of the *φ*. By combining these symmetry elements *χ* and the local field *E_loc_*, the simulated curves shown in Figure 2 can be obtained.

We have also observed the sample rotation angle dependence of the THG from the NC-MC sample at a 45° incidence, as shown in Figure 1b. Figure 2a illustrates the THG intensity from the Ni NC-MCs for the *s*-in/*s*-out combination, as a function of the *φ*. The curve simulated in the previous section is patterned in Figure 2. In fact, the THG in the *s*-in/*s*-out combination showed an isotropic pattern. According to the literature [9], this enhancement is mainly caused by the LSPs from nanostructures on the microcubes. As a consequence, the THG intensity pattern for the *s*-in/*s*-out combination does not depend on the *φ*, and the THG intensity is sensitive to LSP effect on the nanostructures rather than depending on the symmetry and LR effects on microstructures. Figure 2b illustrates the SHG emission intensity in the *s*-in/*s*-out combination, as a function of the *φ* from the Ni NC-MCs [14]. The SHG pattern in the *s*-in/*s*-out polarization configuration exhibits eight dim minima at *φ* = 0°, 45°, 90°, 135°, 180°, 225°, 270°, and 315° [14]. Thus, the SHG intensity pattern for the *s*-in/*s*-out polarization combination strongly depends on the rotation angle *φ*, and the SHG intensity is sensitive to symmetry and LR effects on the microstructures rather than the LSPs’ effect on the nanostructures [14].

Figure 2 shows that there is a large difference between second- and third- harmonic patterns. We hypothesize that the main reason is the magnitude of the contribution of the nanostructures to the LSPs. As Equation (1) points out, only the local field *E_Y,loc_* (including Fresnel factor *F_Y_*(*ω*)) relates to the plasmon excitation if we exclude Fresnel factors *F_Y_*(3*ω*) and *F_Y_*(2*ω*). Due to their weak off-resonance coupling to the LR effect with nanostructures, the field relation would be |*E^LSP^_Y,loc_*| > |*E^LR^_Y,loc_*|. Since the THG signal intensity *I_s_*(3*ω*) depends on the sixth power of the local field |*E_Y,loc_*|^6^, it will receive a contribution from the nanostructures that is more significant than in the case of SHG, depending on the fourth power of the local field |*E_Y,loc_*|^4^.

**Figure 3:**
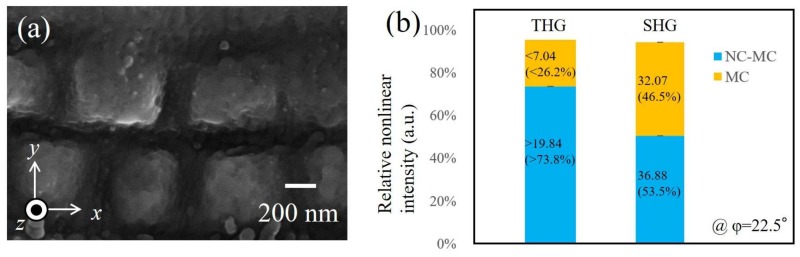
(**a**) SEM image of the microcube (MC) surface; (**b**) relative nonlinear optical intensities for *s*-in/*s*-out at *φ* = 22.5° for NC-MCs and MCs, respectively.

In order to control THG enhancement, we measured the THG signal using the MC sample. In Figure 1a, many nanodots were ablated from the MCs, leading to the isotropic THG as seen in Figure 2a. The SEM image of the surface of the MC sample is shown in Figure 3a. Indeed, several nanodots on microcubes were removed. Figure 3b shows the relative THG and SHG intensities in the *s*-in/*s*-out combination for NC-MCs and MCs on the Ni at *φ* = 22.5°. According to the data, the THG intensity decreases to a greater degree than the SHG intensity. The reason for this is related to the different magnitudes of contribution of THG and SHG to LSPs, as mentioned above.

We describe here the experimental studies for the different magnitudes of the contribution of the THG and SHG to LSPs. Microcube structures created via the laser-ablation method do not have sharper structure at their corners (as seen in Figure 1a), and they are like “dorm-style tents”. Due to this, the magnitude of the LR effect on the cubes should be 10 times weaker than for the perfect cubes [19]. On the other hand, a lot of nanostructures generated via ablation lead to a large LSP effect, as expected [16]. Namely, the magnitude of the electric field enhanced by the LR on the microstructure might be weaker than that enhanced by LSPs on nanostructures. If so, LSP enhancement should result in greater SHG than LR enhancement. However, the magnitude of enhancements cannot be distinguished in Figure 2b. LSP enhancement resulted in greater THG than LR enhancement, as seen in Figure 2a. This is because THG intensity is proportional to the sixth power of electric fields, and thus the difference between their two effects appeared significantly. These facts suggest that LSP-enhanced THG screened LR-enhanced THG. In short, the gap may widen even more if higher-order nonlinear processes are taken into account when the module of the local field on the nanostructures (*E^LSP^_Y,loc_*) is greater than module of the local field on the microcubes (*E^LR^_Y,loc_*) (i.e., |*E^LSP^_Y,loc_*| > |*E^LR^_Y,loc_*|). THG intensity is resonantly enhanced by the LSPs from the nanostructures, and the enhancement due to the LR effect from the microstructures is somehow hidden. These facts show a clear relation between LSP and LR enhancements on the nano/micro structure.

The nano/micro hierarchical structure consisting of differently-sized structures can induce cascaded plasmon field enhancement [14,22]. If the nanostructures are in contact with other nanostructures (as seen in Figure 1a), a large enhancement of the local field *E_Y,loc_*(*ω*) will be obtained thanks to the cascading *E-*field enhancement effect [22] due to the contentious LSP effect. Then, the *E*-field enhancement occurring due to the cascading effect may boost the THG intensity isotopically at the *φ,* as seen in Figure 2a.

## 4. Conclusions

We investigated the THG signal intensity from NC-MC nano/micro structures on Ni. The influence of LSPs on the nanostructures clearly enhanced THG. By removing the nanostructures on the hierarchical structures, we could extract the THG supported by the symmetry and LR effects on microstructures. We found that the enhancement due to the LR effect from the microstructures was hidden by resonant enhancement due to the LSPs from the nanostructures. These interesting physical phenomena could have applications in nonlinear plasmonic devices.
